# Effects of a Peer-Led Intervention on HIV Care Continuum Outcomes Among Contacts of Children, Adolescents, and Young Adults Living With HIV in Zimbabwe

**DOI:** 10.9745/GHSP-D-19-00210

**Published:** 2019-12-23

**Authors:** Talent Tapera, Nicola Willis, Kudakwashe Madzeke, Tanyaradzwa Napei, Mather Mawodzeke, Stanley Chamoko, Abigail Mutsinze, Teddy Zvirawa, Beatrice Dupwa, Aveneni Mangombe, Anesu Chimwaza, Talent M. Makoni, Winnie Mandewo, Mbazi Senkoro, Philip Owiti, Jaya Prasad Tripathy, Ajay M.V. Kumar

**Affiliations:** a Africaid Zvandiri, Harare, Zimbabwe.; b Ministry of Health and Child Care, Harare, Zimbabwe.; c Elizabeth Glaser Pediatric AIDS Foundation, Harare, Zimbabwe.; d National Institute for Medical Research, Muhimbili Centre, Dar es Salaam, Tanzania.; e International Union Against Tuberculosis and Lung Disease, Paris, France.; f National Tuberculosis, Leprosy and Lung Disease Program, Nairobi, Kenya.; g International Union Against Tuberculosis and Lung Disease, South-East Asia Office, New Delhi, India.; h All India Institute of Medical Sciences, Nagpur, India.; i Yenepoya Medical College, Yenepoya, Mangaluru, India.

## Abstract

**Background:**

Africaid Zvandiri, in partnership with the Ministry of Health and Child Care (MOHCC) in Zimbabwe, implemented a comprehensive, peer-led program, focused on children, adolescents, and young adults living with HIV aged 0–24 years. The peers, known as community adolescent treatment supporters (CATS), are people living with HIV (PLHIV) aged 18–24 years who are trained and mentored to support their peers throughout the HIV care continuum through support groups, home visits, phone call reminders, and messages. We report the HIV care continuum outcomes (HIV testing uptake, antiretroviral therapy [ART] uptake, retention, and viral suppression) in a cohort of household contacts and sexual partners (aged younger than 25 years) of index children, adolescents, and young adults living with HIV identified by CATS from October 2017 to September 2018 in 24 districts of Zimbabwe.

**Methods:**

This was a retrospective cohort study involving analysis of routine program data, extracted from electronic databases consisting of data on contacts of index PLHIV and ART outcomes. We used April 30, 2019, as the censor date for all analyses.

**Results:**

A total of 15,223 household contacts and sexual partners with unknown HIV status (linked to 9,353 index PLHIV) were identified and referred for HIV testing. Of these, 12,114 (79.6%) were tested and 1,193 (9.8%) were HIV-positive. Of the latter, 1,153 (96.6%) were initiated on ART with 99% starting on the day of diagnosis. Of those on ART, 1,151 (99.8%) were alive on ART at 6 months and 2 (0.2%) died. A total of 1,044 (91%) children, adolescents, and young people living with HIV underwent viral load testing at 6 months or later, of whom 1,037 (99.3%) were virally suppressed (<1000 copies/ml).

**Conclusion:**

These findings add to the global evidence demonstrating the effectiveness of peer-led interventions in children, adolescents, and young adults living with HIV and justify the decision of the MOHCC in Zimbabwe to scale-up the model nationally. Future research should aim to understand the reasons for the gaps in HIV testing and viral load testing using qualitative research.

## Abstract (precis)

An intervention focused on children, adolescents, and young adults living with HIV using a cadre of dedicated peers—community adolescent treatment supporters—led to improvements along the HIV care cascade among their household contacts and sexual partners.

## INTRODUCTION

Since the first HIV/AIDS patient was reported more than 35 years ago, approximately 78 million people globally have become infected with HIV and 35 million have died from AIDS-related illnesses.^1^ By the end of 2017, there were 36.9 million people living with HIV (PLHIV), of whom 5.7 million (15%) were children (0–9 years), adolescents (10–19 years), and young adults (20–24 years of age).^1^

Adolescents have an increased tendency for risk-taking behavior, including unsafe sexual practices and substance abuse that increases their vulnerability to acquiring HIV.^2^^,^^3^ Apart from the risk behaviors, some children and adolescents have prenatally acquired HIV given the natural history of untreated HIV infections. Between 2005 and 2012, global HIV-related deaths increased among adolescents by 50% but declined among all PLHIV.^4^ Since then, although deaths among adolescents with HIV have been decreasing due to massive scale-up of antiretroviral therapy (ART), the rate of decrease has been slower compared to the rate of decrease among adults.^1^

Several studies in sub-Saharan Africa have shown that, compared to adults, adolescents were less likely to access HIV testing and treatment services, remain in care, and achieve viral suppression.^5^^–^^11^ These results were mainly due to poor prioritization of adolescents in most national HIV plans, inadequate provision of HIV testing and treatment services, delays in diagnosis and treatment, and lack of services to support retention in care.^12^ Children also remain a vulnerable and neglected group with issues related to consent, access, acceptability, cultural norms, stigma, and discrimination.^13^ HIV testing and ART coverage among children are often lower than among adults.^1^

Compared to adults, adolescents are less likely to access HIV testing and treatment services, remain in care, and achieve viral suppression.

Zimbabwe has been facing a generalized HIV epidemic with 1.3 million PLHIV (17% aged younger than 25 years) by the end of 2017.^1^ A national survey conducted in 2015–2016 showed that only 34% of young adults (15–24 years) knew their HIV status compared to 74% among adults,^14^ a figure well below the global UNAIDS 90-90-90 target. Because HIV testing is the gateway to care and treatment, the World Health Organization (WHO) recommends that all the adolescents in settings with generalized epidemics be offered HIV testing and counseling, preferably using community-based approaches including home-based HIV testing and self-testing.^12^ The HIV care continuum begins with diagnosis to linkage to ART treatment to being retained in care to viral suppression.

Since 2002, Africaid Zvandiri, in partnership with the Ministry of Health and Child Care (MOHCC) in Zimbabwe, has been implementing the Zvandiri program. This program is a comprehensive, multicomponent, multidonor funded, differentiated service delivery program focused on the overall development of children, adolescents, and young adults living with HIV that includes HIV care, sexual and reproductive health, mental health, and social protection. At the heart of this program are community adolescent treatment supporters (CATS), adolescents and young adults aged 18–24 years who are living with HIV and who have been trained, mentored, supervised, and incentivized to counsel and offer support to their peers throughout the HIV care continuum, through support groups, home visits, counseling, short message service (SMS), and phone call reminders. Previous program evaluation indicated increased linkage of children, adolescents, and young adults to HIV treatment and retention as well.^15^ Encouraged by these positive results, the MOHCC in Zimbabwe scaled up the CATS model in 51 districts (of 63 in Zimbabwe) in 2017.^15^

Community adolescent treatment supporters have been trained, mentored, supervised, and incentivized to counsel and support their peers throughout the HIV care continuum.

The role of CATS was expanded in 2016 to include contact investigation, including counseling all the household contacts and sexual partners of index HIV patients, referring them for HIV testing, and linking those diagnosed as HIV-positive to care and support including ART. However, there has not been a systematic assessment of how well this expansion is functioning in routine program settings.

Hence, we undertook an operational research study to assess the effect on HIV care cascade outcomes (HIV testing uptake, ART uptake, ART retention, and viral suppression) in a cohort of household contacts and sexual partners (aged younger than 25 years) of index children, adolescent, and young adults living with HIV identified by CATS from October 2017 to September 2018 in selected districts of Zimbabwe.

## METHODS

### Study Design

This was a retrospective cohort study involving analysis of secondary data routinely available in records of the Zvandiri program from October 2017 to September 2018. The study was done from January 2019 to May 2019.

### Setting

Zimbabwe, a landlocked country situated in southern Africa, has a population of 13.1 million. According to a national survey, 74.2% of PLHIV aged 15–64 years knew their HIV status, 86.8% of those who knew their HIV status received ART, and 86.5% of the latter were virally suppressed.^14^

### The Zvandiri Model

**FIGURE 1.**
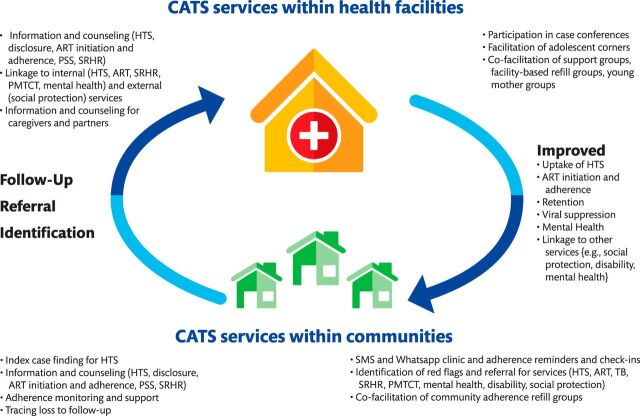
The Zvandiri Model of Care Involving the Community Adolescent Treatment Supporters in Zimbabwe Abbreviations: ART, antiretroviral therapy; CATS, community adolescent treatment supporters; HTS, HIV testing services; PLHIV, people living with HIV; PMTCT, prevention of mother-to-child transmission of HIV; PSS, psychosocial support; SRHR, sexual and reproductive health and rights; SMS, short message service; TB, tuberculosis.

The goal of Zvandiri (meaning “as I am”) program is to achieve and maintain physical, social, and mental well-being of children, adolescents, and young adults living with HIV. CATS are at the forefront of service delivery in this program. The CATS’ major responsibilities in working with children, adolescents, and young adults living with HIV include: (1) cofacilitating monthly support groups and ART refill groups, (2) conducting home visits, (3) sending SMS reminders and check-ins, (4) counseling, (5) making phone calls, (6) referring and linking them to care, (7) conducting community outreach visits, and (8) cofacilitating caregiver workshops. For younger age groups, the CATS support the children through the parents or caregivers. The CATS are incentivized with a fixed allowance of US$20 per month, bicycles to facilitate home visits and/or reimbursement of bus fare, and monthly airtime allowance for SMS reminders and calls. More details about CATS are summarized in Table 1. The model of care has been described in detail elsewhere and is visually summarized in Figure 1.^15^

**TABLE 1.** Description of Community Adolescent Treatment Supporters in the Africaid Zvandiri Program in Zimbabwe

**Who are CATS?**	Adolescent and young adults living with HIV (18–24 years old) trained and mentored by MOHCC and Africaid as peer counselors
**Who appoints CATS?**	Health care facility staff identify PLHIV (18–24 years old) with the potential (willing, competent, and motivated) to be CATS and then appoint them in consultation with the authorities in the MOHCC and Africaid. CATS should have completed secondary school and must have consent from their caregivers to enroll.
**How many CATS per health facility?**	Although the number of CATS per health facility depends on the number of children, adolescents, and young adults living with HIV who need support, the aim is to at least have 1 male and 1 female CATS per health facility. Each CATS should support between 30 and 60 children, adolescents, and young adults living with HIV at any given point in time.
**What training do they receive?**	All CATS receive 2 weeks of MOHCC-endorsed training on knowledge related to pediatric and adolescent HIV (HIV, ART, adherence support, disclosure, sexual and reproductive health, protection, psychosocial support, and mental health) and skills in counseling and community outreach. The training combines theory and practical components, which includes hands-on mentorship (‘shadowing’) by senior CATS for a period of time before they are independently able to provide support. Training is participatory and uses case studies and role plays. They also receive technical support from district-based Zvandiri mentors employed by Africaid. This initial training is then followed by continued on-site training and mentorship.
**What are their responsibilities?**	Cofacilitate monthly support groups and ART refill groups Conduct home visits for counseling, monitoring, and support Send SMS reminders and phone calls for adherence and clinic visits and check-ins Provide counseling in clinic and link to other services as needed Refer children, adolescents, and young adults living with HIV (particularly severe cases) and link to other service providers including OI/ART, mental health, social protection, disability, SRHR, and PMTCT Perform community outreach visits in partnership with other cadres from health and social protection Cofacilitate caregiver workshops
**Who supervises and mentors CATS?**	A nurse or primary counselor at the clinic supervises CATS with additional supervision and mentorship by the district Zvandiri mentor. A district-level monthly meeting is conducted to mentor and review progress of CATS.
**What remuneration and incentives do they receive?**	Fixed allowance of US$20 per month Bicycles to facilitate home visits and/or reimbursement of bus fare Monthly airtime allowance for SMS reminders and calls

Abbreviations: ART, antiretroviral therapy; CATS, community adolescent treatment supporter; MOHCC, Ministry of Health and Child Care; OI, opportunistic infection; PLHIV, people living with HIV; PMTCT, prevention of mother-to-child transmission of HIV; SMS, short messaging service; SRHR, sexual and reproductive health and rights.

Through the CATS’ support of index PLHIV, they had an avenue to meet the index cases’ contacts. The CATS screened the contacts against the age, biological relations, sexual relationship, and prior testing eligibility parameters. They first sought permission of the contacts during home visits and ensured privacy. Through this screening process, CATS referred sexual partners (aged younger than 25 years) as well as household contacts (aged younger than 25 years) who were biologically related and staying together under the same roof for HIV testing and supported pre- and post-test HIV counseling and disclosure. The contact tracing forms were returned and kept in a lockable cabinet at the health facility. There were 3 options provided to the contacts for HIV testing: facility-based testing, self-testing, and home-based testing. Those who opted for self-testing were provided with self-testing kits and, if found to be reactive, they were referred for confirmatory HIV testing at the nearest health facility. If contacts or partners felt hesitant to come to a health facility, home-based testing was done by roving testers from other organizations. The CATS used a standard MOHCC tool to refer the contacts or partners for HIV testing.

Through the CATS’ support of index PLHIV, they had an avenue to meet the index cases’ household contacts and sexual partners.

Contacts and partners found to be HIV-negative were linked to HIV prevention services, including voluntary medical male circumcision, family planning, and cervical cancer screening for young women. Contacts confirmed as HIV-positive were registered in the health facilities for initiating ART. Diagnosis and treatment were conducted per national guidelines, which followed a “test and treat” policy, in line with the WHO guidelines.^16^^,^^17^ In addition to the care contacts and partners received as part of the national program, they were also registered with the Zvandiri program for treatment adherence support and other activities with CATS.

### Differentiated Care

Recognizing that not all children, adolescents, and young adults living with HIV required the same level of care, CATS differentiated and tailored services according to the person’s clinical, psychological, and social needs and circumstances. The 2 types of support, standard support and enhanced support, are summarized in Table 2.

**TABLE 2.** Components and Levels of Support Provided by CATS to Children, Adolescents, and Young Adults Living With HIV in the Africaid Zvandiri Program, Zimbabwe

	**Standard Zvandiri Support**	**Enhanced Zvandiri Support**
**Eligibility criteria**	Undetectable viral load or CD4 count >500 cells/ml in the last 6 months Attended all scheduled clinic visits in the last 3 months Psychologically stable Safe	A detectable viral load or CD4 count <500 cells/ml in the last 6 months Failed to attend scheduled clinic visits in the last 3 months Psychological distress Abuse or neglect Started ART in the past 3 months Reported nonadherence Pregnant
**CATS-led interventions**	Monthly home visit Weekly SMS reminders Clinic-based counseling Referrals and linkages, particularly for severe cases, to other service providers	Home visit every 2 weeks Daily SMS reminders Clinic-based counseling Referrals and linkages, particularly for severe cases, to other service providers
**CATS-supported interventions**	Monthly support group Caregiver workshop	Monthly support group Caregiver workshop Adherence workshop Community outreach with CHWs/CCCWs

Abbreviations: CATS, community adolescent treatment supporters; CHWs, community health workers; CCCWs, child case care workers.

Briefly, CATS provided standard support to PLHIV who were clinically and psychosocially stable and who regularly attended their clinic visits. CATS provided enhanced support to those who did not regularly attend their clinic visits, were not virologically suppressed, or had special needs (e.g., mental health conditions, pregnant women, and those at risk of or subjected to abuse or neglect). In enhanced support, CATS increased the frequency and intensity of contact with children, adolescents, and young adults with HIV. CATS provided counseling and referred severe cases to the Zvandiri mentors and health facility and other relevant service providers.

### Monitoring and Supervision

CATS were attached to the health facilities in their areas. CATS were supervised and supported by the MOHCC staff and district-based Zvandiri mentors employed by Africaid. CATS were expected to submit a monthly report in the prescribed format and attend a monthly review meeting chaired by the Zvandiri mentor. This meeting acted as a forum to discuss successes and challenges and decide on the course-corrective actions.

### Recording

When CATS visited the index cases and contacts at their homes, they captured the information with respect to HIV testing in a paper-based, structured proforma called an index case form. Clients with previously known HIV status were not listed in the index form. This form was digitized in a Microsoft Excel sheet at the district level. The details of contacts diagnosed as HIV-positive and started on ART were then captured electronically in the ART database, each getting a unique identification code (UIC).

### Study Population and Period

The study population comprised household contacts aged younger than 25 years (defined as people who were biologically related and staying together under the same roof) and sexual partners (aged younger than 25 years) of the index PLHIV, identified by CATS from October 2017 to September 2018 in 24 selected districts of Zimbabwe. These districts were purposively chosen based on availability of data (of contacts) in the electronic form. People with a previously known HIV status were not included.

### Data Collection

Data variables included age, sex, HIV testing (yes/no), HIV test result, ART start (yes/no), ART outcomes (alive and on ART/death/loss to follow-up) at 6 and 12 months of care, viral load test, and test result. Data were sourced from 2 databases: (1) contact database, containing the details of the contacts and HIV testing, and (2) ART database, containing the details of ART outcomes and viral load testing. For each HIV-positive client identified in the contact database, we searched the ART database using the UIC, name, and sex to assess if the client has been initiated on ART. This merged database was used for all analysis. The censor date for assessing all the outcomes was April 30, 2019, when the data were downloaded from the ART database.

### Data Analysis

Data analysis was done using EpiData (v2.2.2.187, EpiData Association, Odense, Denmark) and STATA (version 12, Texas, USA) software. HIV testing, HIV positivity, ART initiation, retention in care, and viral suppression (defined as <1000 copies per ml) were summarized using frequencies and proportions. The operational definitions of the outcomes for children, adolescents, and young adults living with HIV were as follows: (1) **Alive and on ART**: those who did not miss their clinical visits and documented to have received care at the time of assessment; (2) **Death**: those who died at any time during the course of treatment; and (3) **Loss to follow-up**: those who were not seen at the ART center for 90 days or more after their scheduled appointment and who could not be contacted successfully.

### Ethical Approval

Ethics approval was obtained from the Medical Research Council of Zimbabwe (MRCZ/E/225) and the Ethics Advisory Group of International Union Against Tuberculosis and Lung Disease, Paris, France (EAG 61/18). Because the study involved a review of existing records without direct interaction with human participants, the need for informed consent was waived by the ethics committees.

## RESULTS

A total of 15,223 household contacts and sexual partners linked to 9,353 index PLHIV (1.6 contacts per index case) were identified by CATS during the study period. There were 278 CATS (approximately 34 index PLHIV per CATS on average) in the study areas. Of the 278 CATS, 155 (55%) were women and 123 were men (45%).

The demographic characteristics of the contacts are shown in Table 3. A majority of the contacts were women (57%). Most of the contacts were either children (57%) or siblings (41%) of the index cases, and the remaining (2%) were sexual partners.

**TABLE 3.** Demographic Characteristics of the Household Contacts and Sexual Partners of Index People Living With HIV Identified by Community Adolescent Treatment Supporters in 24 Districts of Zimbabwe, October 2017–September 2018 (N=15,523)

**Characteristics**	**No. (%)**
**Age** (years)	
0–4	2495 (16.4)
5–9	2814(18.5)
10–14	3170 (20.8)
15–19	3830 (25.2)
20–24	2914 (19.1)
**Gender**	
Male	6597 (43.3)
Female	8626 (56.7)
**Type of Contact**	
Sibling	6229 (40.9)
Sexual partner	290 (1.9)
Children	8704 (57.2)
**Province**	
Bulawayo	1567 (10.3)
Harare	685 (4.5)
Manicaland	4489 (29.5)
Mashonaland Central	1076 (7.1)
Mashonaland East	179 (1.2)
Masvingo	1362 (8.9)
Matabeleland South	1871 (12.3)
Midlands	3994 (26.2)

### HIV Testing

All the 15,223 contacts and sexual partners were referred for HIV testing by the CATS. Of these, 12,114 (79.6%) were tested for HIV, and 1,193 (9.8%) were found to have HIV infection (Table 4). Most (98%) of the contacts were tested for HIV at a health facility and the rest underwent self-testing or home-based testing. In multivariable analysis, age and province were independently associated with not testing for HIV (Table 5). Not testing was significantly higher among younger age groups (i.e., those aged 0–4 years and 5–9 years) when compared to the 20–24 years age group.

**TABLE 4.** HIV Care Cascade Among Household Contacts and Sexual Partners of Index PLHIV Identified by Community Adolescent Treatment Supporters in 24 Districts of Zimbabwe, October 2017–September 2018 (N=15,223)

	**No. (%)**
**HIV Testing Outcome**	
Contacts Referred for HIV Testing	15,223 (100.0)
Contacts tested for HIV	12,114 (79.6)
Contacts tested who were HIV-positive	1,193 (9.8)
HIV-positive contacts who initiated ART	1,153 (96.6)
**ART Outcomes**	
*3 months*	
Alive on ART	1,153 (100.0)
*6 months*	
Alive on ART	1,151 (99.8)
Died	2 (0.2)
*12 Months (n=569* ^a^ *)*	
Alive on ART	566 (99.5)
Died	2 (0.4)
Lost to follow-up	1 (0.2)
Viral suppression (<1000 copies/ml)	1,037^b^ (99.3)

Abbreviations: ART, antiretroviral therapy; PLHIV, people living with HIV.

a Number of contacts eligible for 12-month assessment; People whose duration between ART start date and censor date was less than 12 months were considered not eligible for assessment.

b Among 1,044 contacts who had a viral load test at 6 months or later after starting ART.

**TABLE 5.** Factors Associated With Not Testing for HIV Among Household Contacts and Sexual Partners of Index PLHIV Identified by Community Adolescent Treatment Supporters in 24 Districts of Zimbabwe, October 2017–September 2018

**Characteristic**	**Total Referred for HIV Testing**	**Not Tested for HIV**	**Crude RR** **(95% CI**^a^**)**	**Adjusted RR^b^** **(95% CI**^a^**)**
	**No.**	**No. (%)**		
**Total**	15223	3109 (20.4)		
**Age (years)**				
0–4	2495	864 (34.6)	13.63 (10.82–17.18)	12.72 (10.01–16.17)
5–9	2814	775 (27.5)	10.84 (8.59–13.68)	10.41 (8.19–13.23)
10–14	3170	867 (27.4)	10.77 (8.54–13.58)	10.20 (8.03–12.96)
15–19	3830	529 (13.8)	5.43 (4.28–6.90)	5.21 (4.08–6.65)
20–24	2914	74 (2.5)	Ref	Ref
**Gender**				
Male	6597	1456 (22.1)	1.15 (1.08–1.22)	1.05 (0.98–1.13)
Female	8626	1653 (19.2)	Ref	Ref
**Type of contact**				
Sibling	6229	1289 (20.7)	Ref	Ref
Sexual partner	290	12 (4.1)	0.19 (0.11–0.34)	0.51 (0.29–0.91)
Children	8704	1808 (20.8)	1.00 (0.94–1.06)	1.04 (0.96–1.12)
**Province**				
Masvingo	1362	55 (4.0)	Ref	Ref
Bulawayo	1567	365 (23.3)	5.76 (4.38–7.58)	7.27 (5.47–9.67)
Harare	685	2 (0.3)	0.07 (0.01–0.29)	0.08 (0.02–0.36)
Manicaland	4489	1321 (29.4)	7.28 (5.60–9.47)	7.36 (5.62–9.65)
Mashonaland Central	1076	75 (7.0)	1.72 (1.23–2.42)	1.73 (1.22–2.45)
Mashonaland East	179	9 (5.0)	1.24 (0.62–2.47)	1.34 (0.66–2.72)
Matabeleland South	1871	812 (43.4)	10.74 (8.25–13.99)	11.84 (9.00–15.57)
Midlands	3994	470 (11.8)	2.91 (2.21–3.82)	3.32 (2.51–4.40)

Abbreviations: CI, confidence interval; PLHIV, people living with HIV; Ref, reference group; RR, risk ratio.

a Factors with confidence intervals not including 1 were statistically significant (*P*<.05).

b Adjusted for age, sex, province, and type of contact.

Of the contacts identified and referred for HIV testing by CATS, nearly 80% were tested.

### ART Linkage

Of the 1,193 HIV-positive contacts identified, 1,153 (96.6%) were initiated on ART. Of the latter, 1,144 (99.2%) were initiated on the same day of testing, and the remaining started within a week.

### ART Outcomes

Of 1,153 contacts who started ART, 1,151 (99.8%) were alive ART at 6 months, and 2 (0.2%) had died. At the time of censoring, many clients had not completed 12 months since starting ART and were not eligible for the 12-month assessment. Of the 569 who were eligible for assessment, 566 (99.5%) were alive and on ART, 2 (0.4%) people had died, and 1 (0.1%) was declared lost to follow-up.

### Viral Suppression

Of 1,153 people who started ART, 1,044 (91%) had a viral load test conducted at 6 months or later and of them, 1,037 (99.3%) were found to be virally suppressed.

## DISCUSSION

This study adds to the growing body of evidence demonstrating the effectiveness of targeted, peer-led, differentiated care delivery models in improving the HIV care outcomes among children, adolescents, and young adults living with PLHIV. The CATS model in Zimbabwe is one such successful intervention. We found high rates of HIV testing (80%), ART uptake (97%), retention in care (99%), viral load testing (90%), and viral suppression (99%) in a large cohort of household contacts and sexual partners of index PLHIV under the care of CATS in Zimbabwe. These are excellent outcomes by any standard and better than those reported nationally in Zimbabwe.^14^A population-based study in Zimbabwe reported that among PLHIV aged 15–24 years, only 50.4% knew their HIV status, 83.7% self-reported receiving ART, and 85.4% were virally suppressed.^14^ In this study, 83.7% of the 15–24 year olds who were referred for testing managed to get tested.

This peer-led care model resulted in high rates of HIV testing, ART uptake, retention in care, viral load testing, and viral suppression.

The good outcomes reported in this study may be attributed to the following reasons: (1) a structured and well-defined CATS model implemented in close collaboration with the MOHCC; (2) systematic training of CATS using a standard training curriculum reinforced by continuous on-the-job mentorship by Africaid mentors; (3) close follow-up of the clients by CATS using home visits, SMS reminders, phone calls, and support groups, which concur with the findings of a cohort study in Tanzania that suggested that providing additional psychosocial support to PLHIV receiving ART can reduce loss to follow-up^18^; (4) supportive supervision and monitoring of CATS by the government health care providers; (5) incentives that included a fixed allowance of US$20 per month, airtime allowance for making phone calls and sending SMS reminders to clients, and a bicycle to make home visits; and (6) nonfinancial incentives and motivators such as recognizing the best-performing CATS during the monthly meetings and providing them an opportunity to travel and mentor other CATS in the neighboring districts.

The major gap in the HIV treatment cascade was at the level of HIV testing, where about 20% of the contacts were not tested for HIV. Although we do not know the exact reasons for this gap, we speculate some reasons based on the program experience and multivariable analysis showing the associations of age and province with HIV testing.

The major gap in the HIV treatment cascade was with HIV testing.

HIV testing coverage was low among younger age groups, with the lowest coverage in under-5 children. This may be related to legal barriers, requiring consent of the parent or caregiver.^19^ The CATS ensured that for children requiring caregiver consent for testing, the caregivers were engaged. Although several steps have been taken in Zimbabwe to address this issue including educating the caregivers through workshops, the gap remains. Current efforts target only caregivers of children, adolescents, and young adults living with HIV after HIV diagnosis.^15^ These efforts need to be expanded, and caregivers of all contacts should be educated. There may be other reasons that include cultural norms dictating that young infants should not be taken outside the house except for receipt of vaccines (not always colocated with early infant diagnosis), transportation barriers, a child having to miss school, and the persistent stigma of HIV/AIDS and fear about discrimination.^13^ The CATS screen out children who may have gone through early infant diagnosis for eligibility of HIV testing referral.

HIV testing was lowest in Matabeleland South province. We hypothesize that this may be because this is a border province and many contacts identified initially may have moved across the border to South Africa before HIV testing. This finding needs further investigation. The uptake of self-testing and home-based testing was low in our study. Strengthening these may have potential to fill the gaps in HIV testing. Also, not testing was lowest among the sexual partners (4.1%) compared to 20.7% for siblings and 20.8% for parents. Thus, the program seems to be successful in reaching out to the sexual partners of HIV-positive adolescents and young people.

A strength of the study was that we had a large sample covering 24 districts (of the total of 63) in Zimbabwe, making the findings more generalizable to other areas implementing the CATS model.

### Program Implications

There are a couple of program implications. First, a system should be instituted to routinely capture the reasons for not testing, non-initiation of ART, and other adverse program outcomes. This will enable periodic assessments of the reasons for the gaps in the HIV care cascade and course correction. Second, we identified some inconsistencies in recording including duplicate records. Data on dates of HIV testing and viral load testing were missing, which would have enabled us to assess the delays involved in the process. These need to be corrected, and measures of data quality assurance and quality control should be put in place.

### Limitations

There were some limitations to the study. First, we did not have a control group in our study, which would have enabled a direct head-to-head comparison. Second, we also did not assess the reasons for the gaps in the cascade of care. Third, we did not collect the data on costs, which would have enabled a cost-effectiveness analysis. These knowledge gaps will be addressed by a cluster randomized trial that is underway.^20^ Fourth, we relied on routine program data, which may have had recording errors. Fifth, there was no information on other sociodemographic and clinical factors associated with not testing. So, there could be some bias due to these unexplained confounders.

## CONCLUSION

In conclusion, we found high levels of HIV testing and care outcomes among a cohort of household contacts and sexual partners of index PLHIV who received care by the CATS in Zimbabwe. Contacts of index cases is an additional component of the CATS program that can reach and benefit children, adolescents, and young adults who are HIV-positive and out of care, expanding the potential impact of CATS. Future assessments should focus on exploring the reasons for the gaps in the HIV cascade using qualitative research.

## Notes

**Peer Reviewed**

**First Published Online:** December 16, 2019

**Funding:** Funding for program implementation was provided by the U.S. President’s Emergency Plan for AIDS Relief through the U.S. Agency for International Development, Centers for Disease Control and Prevention, International Training and Education Center for Health, and the United Kingdom Department for International Development.
